# Wnt Inhibitors and Bone Turnover Markers in Patients With Polymyalgia Rheumatica and Acute Effects of Glucocorticoid Treatment

**DOI:** 10.3389/fmed.2020.00551

**Published:** 2020-09-09

**Authors:** Angelo Fassio, Giovanni Adami, Luca Idolazzi, Alessandro Giollo, Ombretta Viapiana, Elisabetta Vantaggiato, Camilla Benini, Maurizio Rossini, Christian Dejaco, Davide Gatti

**Affiliations:** ^1^Rheumatology Unit, University of Verona, Verona, Italy; ^2^Rheumatology Service, South Tyrolean Health Trust, Hospital of Bruneck, Bruneck, Italy; ^3^Department of Rheumatology, Medical University Graz, Graz, Austria

**Keywords:** polymyalgia rheumatica, glucocorticoid, Wnt pathway, Dkk-1, sclerostin, osteoporosis

## Abstract

**Background:** In polymyalgia rheumatica (PMR), data on bone turnover markers (BTM), on Wnt inhibitors (Dkk-1, sclerostin) and their changes induced by glucocorticoids (GC) are lacking. The aims of our study were to compare the baseline levels of Wnt inhibitors and BTM in PMR patients with healthy controls (HC) and to study their changes over the first 4 weeks of GC treatment.

**Materials and Methods:** We enrolled 17 treatment-naïve patients affected by PMR and 17 age and sex-matched healthy controls (HC) from retired hospital personnel. PMR patients were administered methylprednisolone 16 mg daily for 4 weeks. Blood samples were taken at baseline and at week 4 for the PMR group, a single sample was taken for HC. N-propeptide of type I collagen (PINP), C-terminal telopeptide of type I collagen (CTX-I), sclerostin, Dkk-1, and C-reactive protein (CRP) were dosed.

**Results:** At baseline, Dkk-1 was significantly higher in the PMR group as compared to HC (*p* = 0.002) while PINP, CTX-I and sclerostin levels were comparable between PMR patients and HC, After 4 weeks of GC treatment we found in the PMR group a decrease of PINP (mean ± SD percentage decrement as compared to baseline −40 ± 18.6%, *p* < 0.001), CTX-I (−23.5 ± 41.3%, *p* = 0.032), Dkk-1 (−22.4 ± 39.6, *p* = 0.033), and sclerostin (−32.49 ± 20.47, *p* < 0.001) as compared to baseline levels.

**Conclusions:** In treatment-naïve PMR, systemic inflammation is associated with a dysregulation of the Wnt system (increased Dkk-1). Within the 1st month, treatment with GC showed noteworthy effects on bone resorption, formation, and on Wnt pathway modulators.

## Introduction

Polymyalgia rheumatica (PMR) is a chronic inflammatory disease affecting older adults that causes pain, stiffness, and inflammation of the shoulder and pelvic girdles (1). It is typically associated with an increase of acute-phase reactants, and glucocorticoids (GC) still represents the mainstay treatment (1). The expected total treatment duration is 9–12 months provided no relapse occurs (2), but long term treatment is sometimes necessary (3). An adequate decalage of corticosteroid dosage, a careful management of the frequent comorbidities, and the appropriate application of recommended prevention strategies for GC related adverse events (e.g., for glucocorticoid-induced osteoporosis), are crucial to maintain long-term quality of life of these patients (4).

Recently the interest in the role of the Wnt canonical pathway and its main inhibitors (Dkk-1 and sclerostin) in rheumatic inflammatory diseases has been growing. The Wnt pathway stimulates bone formation by fostering osteoblast activity, while it inhibits osteoclastic bone resorption by downregulating the production of Receptor activator of nuclear factor kappa-B ligand (RANKL) (5, 6). The regulation of the canonical Wnt pathway seems to be mainly driven by cytokines acting as potent antagonist. The most important of them are sclerostin and Dickkopf-related protein 1 (Dkk-1) (6, 7).

A dysregulation of the Wnt system has been observed in Rheumatoid Arthritis (RA) (8–11), a chronic inflammatory disease that characterized by generalized and local bone loss (12). Indeed, in RA, pro-inflammatory cytokines such as Tumor Necrosis Factor α (TNFα) and interleukin-6 (IL-6) play a fundamental role in the pathogenesis of inflammation and bone loss, not only enhancing osteoclastic bone resorption, but also limiting bone formation and repair (12). This imbalance is determined by an inappropriate inhibition of the Wnt signaling pathway, mainly due to the overexpression of Dkk-1 which is induced by TNF α in synovial fibroblasts (12, 13).

In addition, other Wnt antagonists such as secreted Frizzled-related protein-1 and sclerostin are induced during inflammation and potentially inhibit repair of bone erosions by suppressing new bone formation (11, 12).

We recently reported that in RA, treatment with GC (leading to a rapid suppression of inflammation) was associated with the decrease in Wnt pathway inhibitors and consequently, with a positive effects on bone resorption markers (11). In the same study, however, we observed also a significant decrease of bone formation marker, despite the decrease in Wnt inhibitors mentioned above (11). So the positive effects of GC due to the inflammation control are unfortunately quickly overtaken by their direct suppression of bone formation and repair capacity (14–16).

Currently, data on bone turnover markers (BTM) in polymyalgia rheumatica are scarce, and data on the profile of Dkk-1, sclerostin, and the changes of bone metabolism induced by GC are completely lacking. The aims of our study were to compare the baseline levels of Wnt inhibitors and BTM in PMR patients with an age matched healthy population and to study their changes over the 1st month of treatment with GC.

## Materials and Methods

We performed a prospective study on consecutive patients affected by PMR classified according to the 2012 EULAR/ACR criteria (17) and evaluated at the rheumatology outpatient clinic of the University of Verona. Exclusion criteria were: (1) treatment with glucocorticoids within the previous 3 months, (2) history of osteoporosis, (3) treatment with bisphosphonates or denosumab within the previous 2 years, or with other drugs known to affect bone metabolism or fracture risk, and/or (4) history of any renal, liver, heart, thyroid, and metabolic bone diseases potentially interfering with the objective of this study. Previous supplementation with cholecalciferol was not an exclusion criterion. Every patient received (if not already taking) 1,000 UI vitamin D and 1 g calcium per day; the dose could not be changed during the whole period of observation. After being diagnosed with PMR, patients were prescribed methylprednisolone 16 mg daily for the first 4 weeks (the duration of the study), according to current recommendations (18). PMR activity score (19) was adopted to assess disease activity at baseline and at week 4.

The time frame of the study was limited to the first 4 weeks because after the first month all patients started an antiresorptive treatment according to national guidelines (20).

The subjects of the healthy controls (HC) group, matched for sex and age, were enrolled from retired hospital personnel. A single blood sample of HC was taken.

The study was conducted within the protocol 1483CESC approved by our local Ethics Committee, in accordance with the 1964 Helsinki declaration and its later amendments or comparable ethical standards. Written informed consent was obtained from all individual participants included.

### Biochemical Analysis

All blood samples were collected in the morning after overnight fasting at baseline and at week 4 for the PMR group, while only a single sample was collected for the HC group. The samples were stored upon collection and at −50°C and finally assayed, in single batch, for N-propeptide of type I collagen (PINP, marker of bone formation), C-terminal telopeptide of type I collagen (CTX-I, marker of bone resorption), sclerostin, Dkk-1, and C-reactive protein (CRP). All the samples were processed in the laboratory of the Rheumatology Unit of the University of Verona. Bone turnover markers (PINP and CTX-I) were measured by the IDS-ISYSMulti-Discipline automated analyzer (Immunodiagnostic System, Boldon, UK) based on chemiluminescence technology. The coefficients of variation (CV) intra-assay measured in our laboratory were 4% for PINP (inter-assay CV 6%), 3% for CTX-I (inter-assay CV 7%). Serum Dkk-1 and sclerostin were measured by ELISA (Biomedica Medizinprodukte, Vienna, Austria) with a sensitivity of 1.7 and 3.2 pmol/L and intra-assay CV of 7 and 5% (inter-assay CV 8.2 and 6.9%), respectively. CRP was measured by ELISA, (DRG Instruments GmbH, Germany), with an inter-assay CV <4.1% and an intra-assay CV <7.5%.

### Statistical Analysis

A statistical power analysis was performed for sample size estimation. The sample size was based on the assumption that a ≥15% decrease in the values of Dkk-1 after 4 weeks of treatment with GC was found. This assumption was based on the data of a previous study in RA (11). We calculated a required sample size as *n* = 14, with an alpha = 0.05 and power = 0.80.

The differences between the values of the different parameters tested between PMR and HC at any observation point were analyzed by *t*-test for normally distributed variables and Mann–Whitney *U*-test for independent samples for non-normally distributed variables.

The differences between the values of the markers at week 4 vs. baseline were analyzed by testing respective percentage changes deltas vs. 0 by one-sample *t*-test. Normality for continuous variables was tested with Shapiro–Wilk test. Two-sided *p*-values of 0.05 or less were considered significant. Data are presented as mean ± SD for normally distributed variables and median [IQR] for non-normally distributed variable.

All analyses are based on a significance level of 0.05. SPSS software, Version 22 (SPSS, Inc., Chicago, IL, USA).

## Results

We enrolled a total of 17 patients affected by PMR (7 males and 10 females) and 17 HC (7 males and 10 females). Median disease duration was 3 [2–4] months. Additional clinical characteristics of PMR patients are summarizes in Table 1. None of the enrolled patients tested positive for rheumatoid factor or anti-citrullinated antibodies and at the physical examination none had evidence of peripheral arthritis.

**Table 1:** Baseline and follow up values of the parameters for healthy controls (HC) and patients with polymyalgia rheumatica (PMR).

	**HC group (*N* = 17)**	**PMR group (*****N*** **=** **18)**	
	**(Single observation)**	**Baseline**	**W4**
Age, mean (SD)	76.3 (8.1)	78.3 (8.6)	/
Gender (M:F)	7:10	7:10	/
BMI mean (SD)	26.1 (2.4)	24.9 (2.4)	/
CRP, median (IQR) mg/L	/	41 (20–51)	3 (3–3.5)^d^
PMR AS	/	30.1 (25.9–33.2)	6.5 (6.3–7.3)^d^
PINP, mean (SD) ng/mL	52.8 (20.8)	59.0 (17.0)	34.4 (12.1)^b^^,^^d^
CTX-I, median (IQR) ng/mL	0.395 (0.287–0.494)	0.340 (0.302–0.45)	0.268 (0.19–0.423)^a^ (*p* = 0.049)^c^
Dkk-1, median (IQR) pmol/L	21.5 (15.2–31.1)	33.6 (29.7–42.0)^b^	23.9 (16.4–40.3)^c^
Sclerostin, mean (SD) pmol/L	44.7 (19.6)	41.9 (15.2)	26.9 (9.2)^b^^,^^d^

a *p < 0.05 vs. HC*.

b *p < 0.01 vs. HC*.

c *p < 0.05 vs. baseline*.

d *p < 0.01 vs. baseline*.

*HC, healthy controls; PMR, polymyalgia rheumatica; W, week; SD, standard deviation; IQR, interquartile range; BMI, body mass index, CRP, C-reactive protein, PMR AS, polymyalgia rheumatica activity score, PINO, N-propeptide of type I collagen; CTX-I, C-terminal telopeptide of type I collagen*.

**Figure 1:**
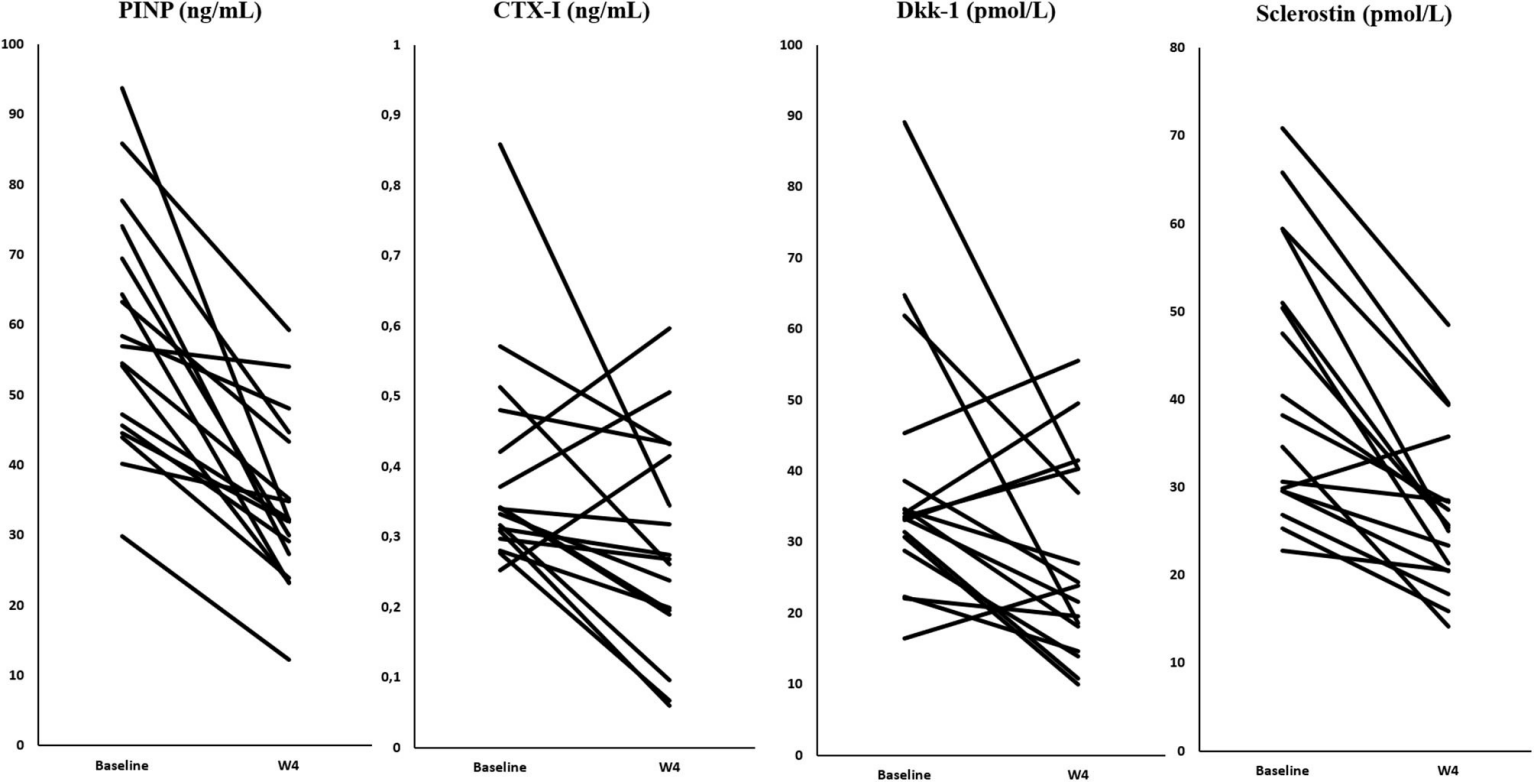
Changes in bone turnover markers, Dkk-1, and sclerostin in the PMR group.

At baseline, only Dkk-1 was significantly higher in the PMR group (*p* = 0.002) than in HC (Table 1). Changes from baseline to week 4 regarding BTM, Dkk-1 and sclerostin for the PMR group are depicted in Figure 1.

After 4 weeks of GC treatment all patients were in clinical remission and CRP values turned to normal. Concerning markers of bone metabolism, we found in the PMR group a decrease of PINP (mean ± SD percentage decrement as compared to baseline −40 ± 18.6%, *p* = 0.000), CTX-I (−23.5 ± 41.3%, *p* = 0.032), Dkk-1 (−22.4 ± 39.6, *p* = 0.033), and sclerostin (−32.49 ± 20.47, *p* < 0.001) as compared to baseline levels.

A significant positive correlation was found between the percentage decrease in CRP and Dkk-1 from baseline to week 4 (*p* = 0.007, *r*_*s*_ = 0.625).

## Discussion

This is the first study that investigated BTM and Wnt inhibitors in patients affected by PMR at baseline and after 4 weeks of treatment with GC.

Our data demonstrate that in treatment-naive PMR patients, Dkk-1 are increased when compared with HC. In addition, the decrease of systemic inflammation after 4 weeks of GCs (all patients were in clinical and laboratory remission at that time) is associated with a decrease in Wnt inhibitors (Dkk-1 and sclerostin), CTX-I, and in PINP.

The finding of increased levels of Dkk-1 in untreated PMR patients is not surprising. Indeed, it is consistent with similar observations in RA, arguably the most common rheumatic inflammatory disease (8, 10, 21). Pro-inflammatory cytokines such TNFα and IL-6 play a fundamental role in the pathogenesis of inflammation and bone loss in RA, partly due to the exaggerated production of Dkk-1 and other Wnt antagonists (12, 13). Consistent with this model, treatments with TNFα inhibitors or GC by reducing pro-inflammatory cytokines in RA patients have been shown to rapidly decrease Dkk-1 (and sclerostin) serum levels (9, 11, 22, 23). Given the central pathogenetic role of TNFα and especially IL-6 in PMR (4) a similar model might be applied to interpret the dysregulation of the Wnt system in this disease, and the reduction of its inhibitors that we observed with CG treatment. The significant positive correlation we found between the decrease of CRP and Dkk-1 seems to support this conclusion.

While the normalization of Dkk-1 after 4 weeks of GC treatment can be explained mainly by the link between inflammation and excessive Wnt inhibition, the reduction of sclerostin even below the levels of HC could be due to the direct effects of GC on this molecule. In a recent study, Thiele et al. (24) reported not only an *in vitro* decrement of sclerostin expression after GC treatment in human bone marrow stromal cells, but also found decreased serum sclerostin in a cross-sectional observation of 21 patients with long lasting (average duration of 1.31 years) PMR who received chronic treatment with GC. Previous studies on autoimmune diseases different from PMR support the presence of contradictory effects of GC on Wnt antagonists (25, 26). It is therefore possible that the suppressive effect of GC on sclerostin expression in PMR could be two-fold: an indirect effect via the inhibition of inflammation (similarly to Dkk-1), and a direct effect, independent from disease activity and inflammation, on cells of the mesenchymal lineage (i.e., osteocytes) (24).

In our study, PINP but also CTX-I yielded a significant decrement after 4 weeks of GCs not only with respect to baseline levels but also with respect to HC, even if at baseline, levels were comparable between patients and HC (a lack of statistical power in this regard can certainly not be excluded). Inflammatory rheumatic conditions can be associated with both local and systemic bone resorption due to the pathologic increases of proinflammatory cytokines and mediators (27). In this case however, levels of CTX-I should have been higher in PMR patients than HC at baseline. Suppression of pathologic inflammation is therefore expected to have positive effects in this setting, both via the suppression of proinflammatory cytokines and via the already discussed reduction in Dkk-1 and sclerostin, with subsequent positive effects on the regulation of the RANKL/OPG ratio (27). However, it should be noted that the absolute serum levels of CTX-I at week 4 were lower than those of HC. In this regard, a relationship between the decrease in bone resorption and the marked suppression of sclerostin might be speculated, albeit a counter regulatory homeostatic response to the decrease in PINP, often seen during treatment with osteoactive drugs (27), cannot be ruled out.

Indeed, we also observed a decrease in PINP with GC that is in line with previous studies (11, 14, 15) and confirms the renown (direct) detrimental effects of GC on osteogenesis and bone formation. Given the suppression of Dkk-1 and sclerostin, the impairment of the bone anabolic activity suggested by the decrease in PINP appears to be largely independent from the Wnt system. Our observations seem to be in line with the concept of a direct “toxic” role of GC toward cells of the osteoblastic lineage and their precursors, resulting in a deficiency in bone forming surfaces (27, 28).

Bone loss is among the main consequences of the RA, and it is well-known to happen both systemically (osteoporosis) and locally (i.e., erosions) (27). RA has been shown to be a risk factor for osteoporosis independently from the treatment with GC (29), also in pre-menopausal women (30). On the other hand, despite the presence of few studies suggesting an increased risk for osteoporosis also in PMR, the overall body of evidence is still insufficient, and the independent contribution of the disease is not clear (31). In addition, local bone loss in PMR is not considered a typical finding. The reason for the different impact on bone health of these two conditions is still unknown. Further data are needed to investigate the balanced relationship between systemic inflammation, pharmacological treatment (GC, anti IL-6, and osteoactive drugs) and generalized and localized bone loss. An in-depth understanding of the differences and similarities of the dysregulation of the Wnt pathway and of other serum biomarkers such as serum osteopontin (32) in inflammatory diseases (i.e., PMR, RA, Giant Cell Arteritis, etc.) and on the dysregulation of the RANKL/OPG ratio may prove useful in the future and contribute to improve the management of these conditions.

Our study has several limitations. First of all, sample size was limited. The sensitivity analysis was calculated on the expected changes of Wnt inhibitors, and therefore it was not powered to rule out, for instance, the absence of differences in BTM and/or sclerostin at baseline. Second, it lacks direct evaluation of bone histology and metabolism. A bone biopsy before and after GC treatment would have been desirable to correlate the findings from serum with actual effects on bone remodeling, however, it was deemed as not being ethical. Long-term observations of patients (e.g., after 6–12 months of GC therapy and in stable remission) would have been of interest to better investigate the direct effects of GCs in PMR on bone metabolism, however, since all patients started anti-resorptive therapy after 1 month of GC therapy (which is in accordance with national guidelines), an unbiased assessment would not have been possible.

In conclusion, this study for the first time showed that, in treatment-naïve PMR, systemic inflammation is associated with a dysregulation of the Wnt system (especially due to the increase in Dkk-1), similarly to what has been observed in RA. Treatment with GC, currently the mainstay therapy for PMR, is associated with suppression of Dkk-1 and sclerostin with a consensual reduction in bone resorption. Nevertheless, CG still show some undesired effects on bone metabolism, namely the suppression of bone formation that seems not to be directly related to the regulation Wnt-pathway.

In PMR a different anti-inflammatory approach such as treatment with IL-6 blockers could enable us to control disease and inflammation limiting the negative consequences of GC on bone health.

## Data Availability Statement

The raw data supporting the conclusions of this article will be made available by the authors, without undue reservation.

## Ethics Statement

The studies involving human participants were reviewed and approved by our local Ethics Committee (Verona,Italy) and the study was conducted within the protocol 1483CESC, in accordance with the 1964 Helsinki declaration and its later amendments or comparable ethical standards. Written informed consent was obtained from all individual participants included. The patients/participants provided their written informed consent to participate in this study.

## Author Contributions

AF and GA drafted the manuscript. LI, AG, OV, EV, and CB collected the data. AF, GA, MR, CD, and DG equally contributed to the interpretation of the data and the revision of the discussion. All authors contributed to the article and approved the submitted version.

## Conflict of Interest

AF reports personal fees from Abiogen, Novartis, Neopharmed, outside the submitted work. LI reports personal fees from Eli-Lilly, Merck Sharp & Dohme, Novartis, Sanofi, Celgene, UCB outside the submitted work. MR reports personal fees from AbbVie, Abiogen, Eli-Lilly, Merck Sharp & Dohme, Novartis, Sanofi, UCB, outside the submitted work. DG reports personal fees from Abiogen, Amgen, JanssenCilag, Mundipharma and Pfizer, outside the submitted work. The remaining authors declare that the research was conducted in the absence of any commercial or financial relationships that could be construed as a potential conflict of interest. The reviewer (EB) declared a past collaboration with one of the authors (CD) to the handling editor.
